# Community Structure, Diversity and Potential of Endophytic Bacteria in the Primitive New Zealand Medicinal Plant *Pseudowintera colorata*

**DOI:** 10.3390/plants9020156

**Published:** 2020-01-27

**Authors:** Neeraj Purushotham, Eirian Jones, Jana Monk, Hayley Ridgway

**Affiliations:** 1 Lincoln University, Department of Pest-Management and Conservation, Lincoln 7647, New Zealand; 2 AsureQuality, Lincoln 7647, New Zealand; 3 The New Zealand Institute for Plant & Food Research Limited, Lincoln 7608, New Zealand

**Keywords:** endophytic bacteria, endophytes, plant-microbe interactions, plant growth promotion, microbial ecology, medicinal plant, Illumina MiSeq

## Abstract

Although the importance of the plant microbiome in commercial plant health has been well established, there are limited studies in native medicinal plants. *Pseudowintera colorata* (horopito) is a native New Zealand medicinal plant recognized for its antimicrobial properties. Denaturing gradient gel electrophoresis (DGGE) and Illumina MiSeq analysis of *P. colorata* plants from ten sites across New Zealand showed that tissue type strongly influenced the diversity and richness of endophytic bacteria (PERMANOVA, *P* < 0.05). In addition, two OTUs belonging to the genus *Pseudomonas* (Greengenes ID: 646549 and 138914) were found to be present in >75% of all *P. colorata* leaf, stem and root samples and were identified as the members of the *P. colorata* “core endomicrobiome”. Culture-independent analysis was complemented by the recovery of 405 endophytic bacteria from the tissues of *P. colorata*. Some of these cultured endophytic bacteria (n = 10) showed high antagonism against four different phytopathogenic fungi tested. The influence of endophytic bacteria on plant growth was assessed by inoculating *P. colorata* seedlings. The mean shoot height of seedlings treated with *Bacillus* sp. TP1LA1B were longer (1.83×), had higher shoot dry weight (1.8×) and produced more internodes (1.8×) compared to the control.

## 1. Introduction

Bacteria are ubiquitous and present in almost all environments, however, their roles within ecosystems and their associations with their hosts are not fully understood. Plants are inhabited by diverse communities of endophytic microorganisms, including endophytic bacteria and fungi which collectively form the “plant endomicrobiome”. Research has demonstrated that endophytic bacteria play crucial roles in plant development by enhancing plant metabolism, improving nutrient uptake, and influence overall fitness [1,2]. Endophytic bacteria include Gram-positive and Gram-negative bacteria from the classes Alpha, Beta, Gammaproteobacteria and Actinobacteria. Endophytic bacteria have been reported to confer several beneficial traits for their hosts such as solubilizing phosphate, assimilating nitrogen and promoting plant growth via the production of phytohormones and growth-regulating enzymes [3]. 

Endophytic bacteria can also mediate biological control of phytopathogens by several mechanisms such as competing for ecological niche, production of bioactive compounds and induced systemic resistance [4]. In addition, endophytes from medicinal plants have been identified as sources of novel antimicrobial compounds [5,6]. Research on the Chinese medicinal plant *Ferula songorica* revealed that the plant was a rich reservoir of endophytic bacteria that were capable of solubilizing phosphate and producing enzymes such as protease and cellulase [7]. Although microbiome research over the last decade has deciphered the complex interactions of the microbial communities with biotic and abiotic factors, there is limited information especially for native medicinal plants. The characterization of endophytic bacteria found in the tissues of native medicinal plants could offer significant insights into the health and ecology of these plant species. As with international examples, New Zealand medicinal plants are likely to host endophytic bacteria with uncharacterized functions [8,9]. 

*Pseudowintera colorata* (horopito) is a slow-growing medicinal shrub found in the sub-alpine regions of New Zealand. There are four species within this endemic genus, belonging to Winteraceae, a primitive family of angiosperms recognized for having structures called tracheids instead of xylem vessels [10]. To date, there are no studies on the community structure of endophytic bacteria inhabiting this primitive terrestrial plant family, which globally comprises approximately 65–90 species across eight genera. Traditional medicine (Rongoā) in New Zealand recognized *P. colorata* for its medicinal properties and as a treatment for ailments such as toothache and skin infections [11]. The leaves of *P. colorata* contain the sesquiterpene dialdehydes polygodial and 9-deoxymuzigadial which have been identified as compounds with strong antifungal, antibacterial and insect antifeedant properties [12,13,14,15]. In addition, polygodial has a very pungent and peppery taste and has also been reported in other plants such as *Polygonum hydropiper* and also in liverworts.

As many microorganisms are not culturable outside their host, molecular tools such as DGGE are common approaches used to study microbial communities [16]. For example, using DGGE in the marine angiosperm *Posidonia oceanica*, it was revealed that the root bacterial communities were significantly different from the communities in rhizomes and leaves [8]. New sequencing technologies such as Illumina have greater depth, detect and identify more species with greater accuracy [17,18]. For example, using amplicon sequencing with Illumina MiSeq, Proteobacteria, Firmicutes, Actinobacteria and Bacteroidetes were found to be the predominant genera in the roots of *Aloe vera* [19]. 

A number of international studies detail the critical importance of the plant microbiome to the ecology and success of the plant, and these microbial associations are also likely to be significant within *P. colorata*. The main objectives of this study were to (i) characterize the community structure and diversity of the *P. colorata* endophytic bacteria using culture-independent techniques such as DGGE and Illumina MiSeq (ii) isolate and identify culturable endophytic bacteria from leaves, stems and roots of *P. colorata* (iii) determine whether the cultured representatives have antimicrobial properties and/or influence the growth of *P. colorata* seedlings. 

## 2. Results

### 2.1. Culture Independent Analysis

#### 2.1.1. Analysis of the Bacterial Endomicrobiome using DGGE

**Figure 1:**
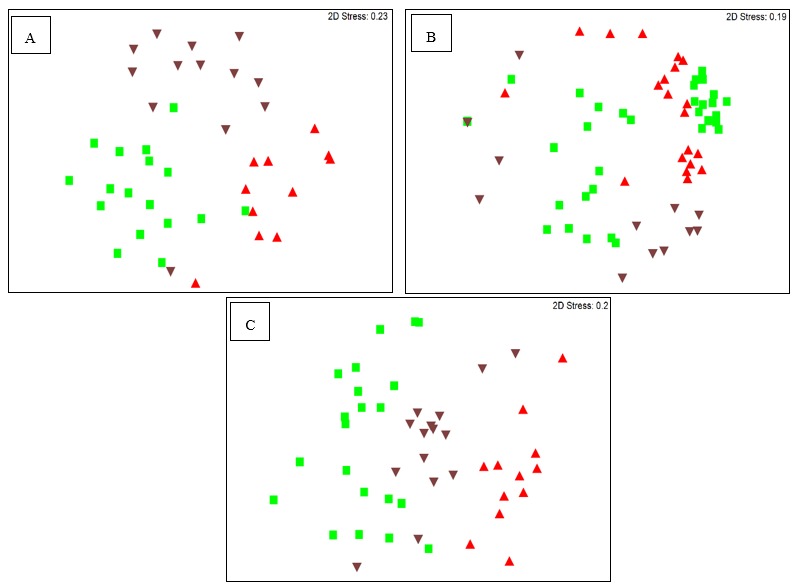
Nonmetric multidimensional scaling (nMDS) plots showing (**A**) Alphaproteobacteria, (**B**) Betaproteobacteria and (**C**) Gammaproteobacteria communities from *P. colorata* leaf (green square), stem (red upright triangle) and root (brown inverted triangle).

According to the DGGE gel patterns and analysis, plant tissues and interaction with location (*n* = 10) influenced the Alpha, Beta and Gammaproteobacteria communities in *P. colorata* (PERMANOVA, *P* ≤ 0.05) (Table 1). Alphaproteobacteria communities in the leaves, stems and root samples formed discrete clusters whereas Gammaproteobacteria communities formed clusters only in the stems and no discernible clustering was observed for Betaproteobacteria communities (Figure 1A–C). A total of 67, 80 and 84 Alpha, Beta and Gammaproteobacteria taxa, respectively, were identified by DGGE. The richness of Alpha, Beta and Gammaproteobacteria was generally higher in leaves (*n* = 17, 22 and 22 respectively) compared to stems (*n* = 17, 16 and 12 respectively) and roots (*n* = 13, 14 and 14 respectively) (least significant difference (LSD) *P* ≤ 0.005) (Table 1) (Tables S1, S2, S3). 

#### 2.1.2. Analyzing the Structure of *P. colorata* Bacterial Endomicrobiome using Illumina MiSeq Metabarcoding 

**Figure 2:**
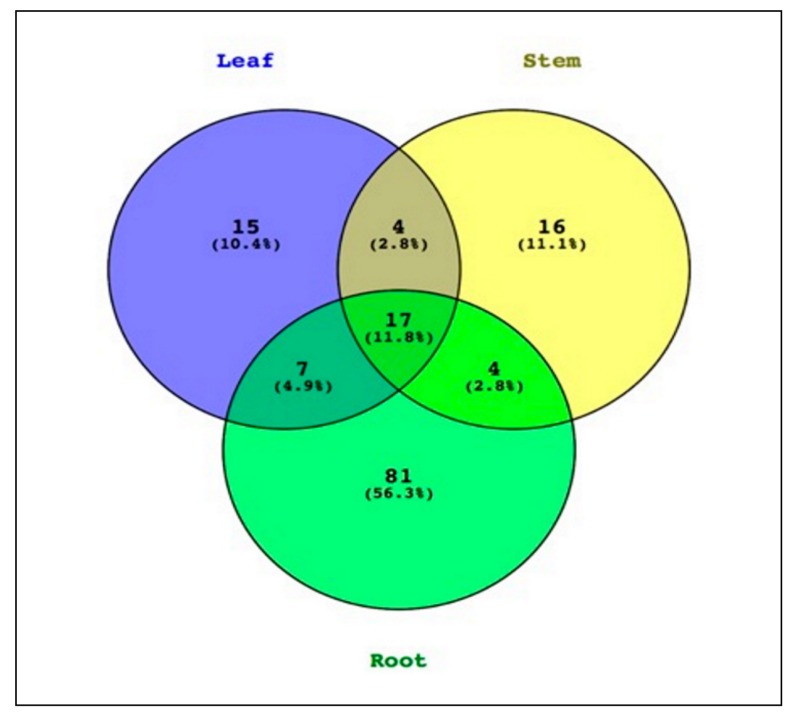
Venn diagram showing endophytic bacteria OTUs in different plant tissues of *Pseudowintera colorata*. The total observed OTUs from QIIME were processed in VENNY (http://bioinfogp.cnb.csic.es/tools/venny/index.html).

An average of 1379 (minimum = 124, maximum = 4308), 3159 (minimum = 185, maximum = 11,501) and 8711 (minimum = 1637, maximum = 20,467) reads were obtained from the leaves, stem and root samples of *P. colorata*, respectively. The reads clustered into 144 OTUs (97% similarity) with an average of 8, 9 and 21 OTUs obtained from leaf, stem and root samples of *P. colorata*, respectively, with some of the OTUs appearing in all tissues. From the non-rarefied data, a total of 11.8% of OTUs were shared between the three tissue types (Figure 2). OTUs that were unique for each of the tissue types accounted for 77.8% of the total OTUs in *P. colorata*. 

**Figure 3:**
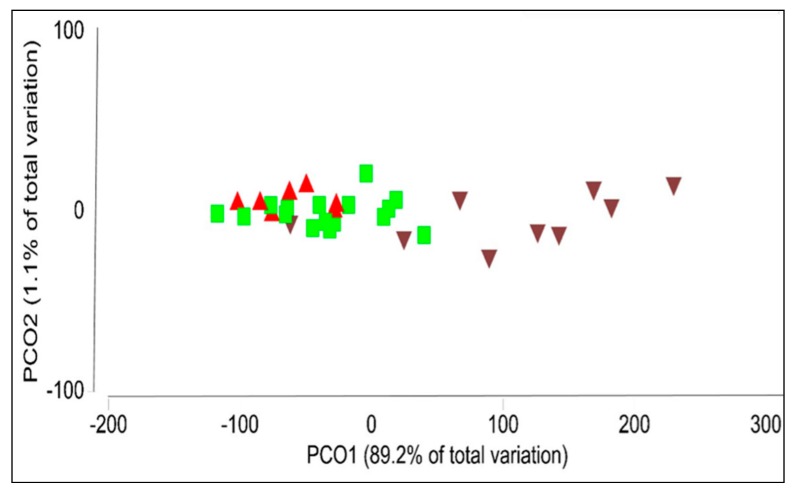
Principal coordinates showing similarities between communities of bacterial endophytes from different tissues in *Pseudowintera colorata*. Leaf: green square; stem: red upright triangle; root: brown inverted triangle.

Tissue type influenced the richness, diversity and community structure of bacterial endophytes in *P. colorata*. The alpha diversity showed differences in bacterial richness between *P. colorata* tissues. The richness differed in above ground (leaf and stem) and below ground (root) tissues (leaf vs. stem, *P* = 0.043; leaf vs. root, *P* = 0.009; stem vs. root, *P* = 0.002). Based on the weighted UniFrac analysis, plant tissue affected the composition of endophytic bacterial communities (PERMANOVA, *P* = 0.001). The bacterial communities clustered based on the plant tissue, with the leaf and stem communities clustering together whereas the root communities being more diverse (Figure 3).

**Figure 4:**
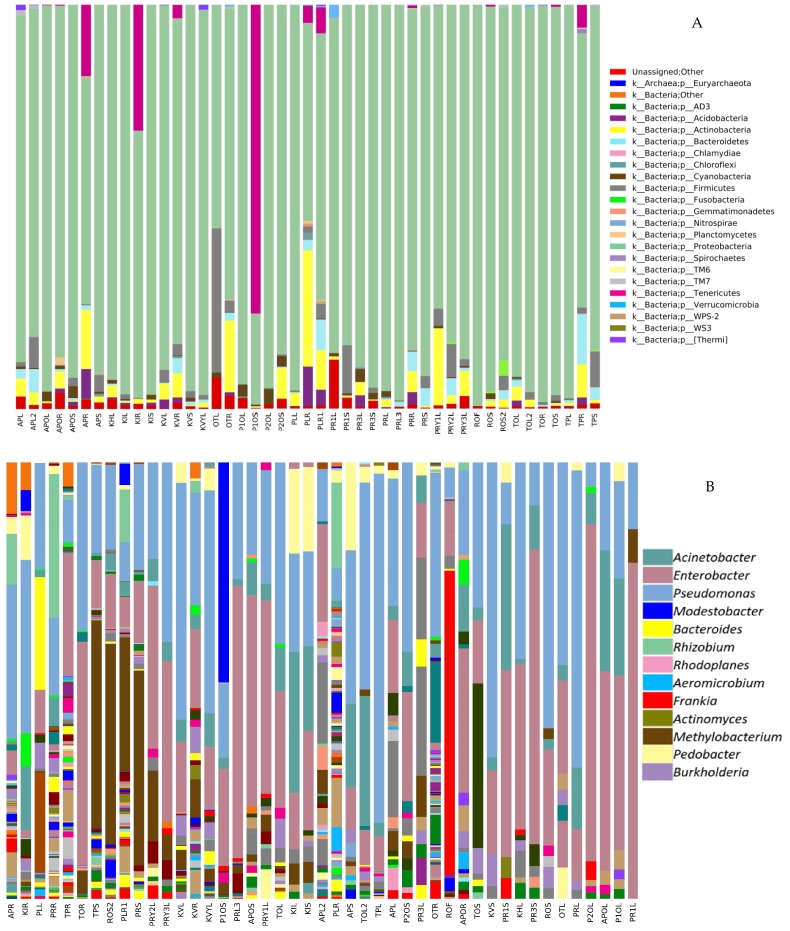
Bar charts showing community structure of endophytic bacteria in different plant tissues of *Pseudowintera colorata* as shown by Illumina MiSeq 16S rRNA amplicon sequencing at (A) the Phylum and (B) Genus level (showing the most dominant genera). Y-axis represents the samples.

The relative abundance of Proteobacteria was high in all tissues (97.6%), followed by Actinobacteria (1.2%), Tenericutes (0.7%), Firmicutes (0.1%), Acidobacteria (0.1%) and Bacteroidetes (0.1%) which were relatively less abundant phyla (Figure 4A). At the class level, Gammaproteobacteria was the most abundant class (89.1%) followed by Alphaproteobacteria (10.0%), Actinobacteria (1.12%) and Betaproteobacteria (0.7%). Less abundant classes were Acidobacteria (0.1%), Bacilli (0.1%), Clostridia (0.05%), Bacteroidia (0.05%) and Saprospirae (0.05%) (data not shown). At the genus level, *Pseudomonas*, *Acinetobacter*, *Methylobacterium*, *Burkholderia*, *Actinomyces* and *Frankia* were some of the most common genera found (Figure 4B). Two OTUs (Greengenes ID: 646549 and 138914) belonging to the genus *Pseudomonas* were found in >75% of all *P. colorata* leaf, stem and root samples and were identified as the members of the *P. colorata* core endomicrobiome.

#### 2.1.3. Prediction of the Function of Endophytic Bacteria in *P. colorata* Using PICRUSt

**Figure 5:**
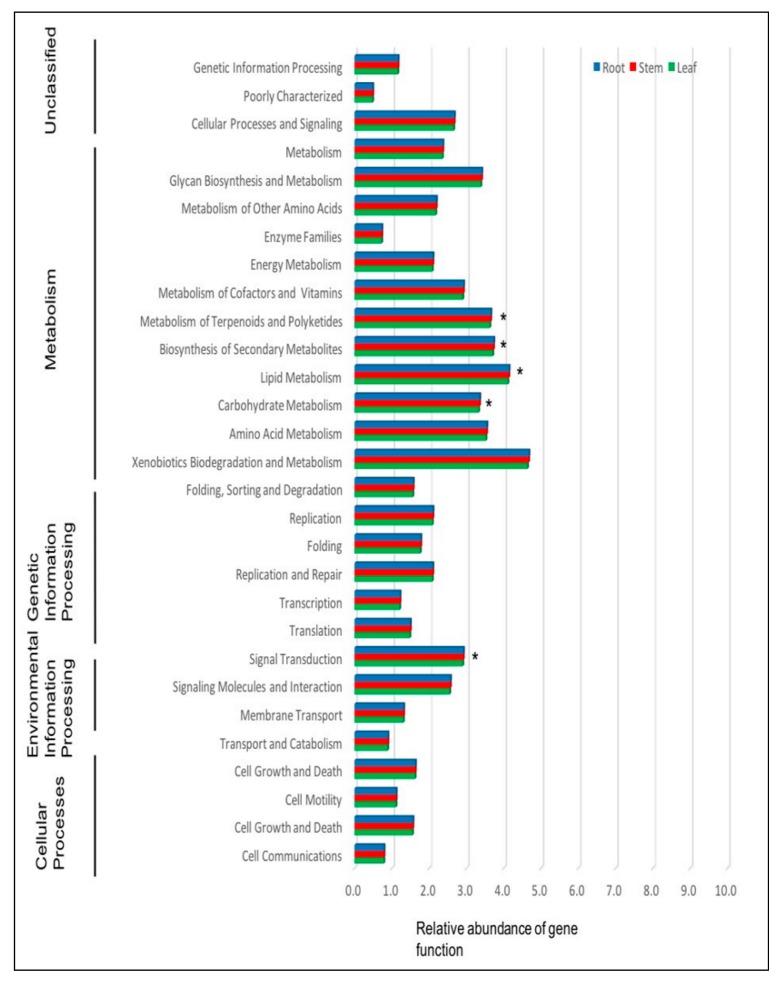
Predicted functions (level 2 KEGG orthology group) of the endophytic bacteria in different plant tissues of *Pseudowintera colorata*. An asterisk indicates gene functions that are significantly different (LSD, *P* < 0.05).

PICRUSt analysis revealed 29 level 2 KEGG orthology groups. Further analysis revealed that 3.6% of the genes in total relative abundance were associated with the biosynthesis of secondary metabolites and that gene functions associated with metabolism of cofactors and vitamins, metabolism of carbohydrates, lipids and amino acids, cell motility and signal transduction were significantly different within the tissues of *P. colorata* (LSD, *P* ≤ 0.05) (Figure 5).

### 2.2. Culture Dependent Analysis

#### 2.2.1. Recovery of Endophytic Bacteria from *P. colorata*

A total of 405 endophytic bacteria were recovered from the surface-sterilized tissues of *P. colorata*. Most of the endophytic bacteria were isolated from the stem (58.1%, *n* = 235), followed by roots (32.1%, *n* = 130) and leaves (9.8%, *n* = 40). No bacteria grew on the plates on which the leaf imprints were taken and the wash water was plated demonstrating that the surface sterilization process was effective. 

#### 2.2.2. Activity of Endophytic Bacteria against Phytopathogenic Fungi

Of the total endophytic bacteria (*n* = 405) tested for activity against four different phytopathogenic fungi, 7 isolates inhibited all the phytopathogenic fungi tested (Table 2). 

#### 2.2.3. Identification of Bioactive Bacteria 

In this study, only isolates that showed high activity (zones of inhibition > 3–7 mm) against test pathogens were selected for identification using 16S rRNA sequencing. Sequencing the PCR products (1500 bp) identified the isolates as *Pseudomonas* (*n* = 4), *Bacillus* (*n* = 4), *Erwinia* (*n* = 1) and *Pantoea* (*n* = 1) (Table 3). 

#### 2.2.4. Influence of Endophytic Bacterial Inoculants on *P. colorata* Seedlings

*P. colorata* seedlings treated with endophytic bacteria showed an increase in the growth for both the treatments in comparison to the control (*P* < 0.05). Seedlings treated with *Pantoea* sp. AP1SA1 had a mean shoot height which was 1.8× longer than the control. Treatment with *Bacillus* sp. TP1BA1B increased the shoot and root dry weight of the seedlings were 1.6× heavier than the control, respectively (Table 4). *Bacillus* sp. TP1LA1B and *Pantoea* sp. AP1SA1 treated seedlings produced 1.8 × more internodes compared to the control.

## 3. Discussion

This is the first study to characterize the structure and diversity of the endophytic bacterial communities in the primitive medicinal plant *P. colorata* using Illumina sequencing. 

DGGE analysis revealed that the composition and richness of bacterial endophytes in *P. colorata* were influenced by tissue type. These results were congruent with previous work showing tissue type as the main factor influencing the similarity and richness of endophytic bacteria in the medicinal plants *Stellera chamaejasme* and *L. scoparium* [20,21]. DGGE analysis revealed that there was overlap (85%) in the Alphaproteobacteria, Betaproteobacteria and Gammaproteobacteria taxa within the three tissue types of *P. colorata*. 

Amplicon sequencing also revealed that root communities included 4.9% and 2.8% of leaf and stem OTUs, respectively, and that 11.8% of the total OTUs were common to all the three tissue types. Roots harbored a large reserve of endophytes (56.3%), which were not shared by other tissues and were specific to roots only. This could be because roots are immersed in the soil and are in constant interaction with rhizosphere microbial communities [22]. In addition, roots are also naturally wounded by insects feeding on them and the emergence of lateral roots which may provide entry points [23]. In this study, the relative richness of the roots may also be attributed to the absence of antimicrobial compounds as only the leaves of *P. colorata* are known to produce polygodial. Several other groups have reported similar findings co-relating the absence of antimicrobial compounds and the relative richness of roots [4,24,25]. 

Gammaproteobacteria class, particularly the genus *Pseudomonas*, was the most relatively abundant group in the endomicrobiome, making up 89.1% of the total reads with the classes Alphaproteobacteria, Actinobacteria and Betaproteobacteria comprising the remaining reads. Two *Pseudomonas* OTUs were identified as members of *P. colorata* core endomicrobiomes as they were present in at least 75% of samples. The definition of the “core endomicrobiome” is variable within the literature with some research groups defining it as the OTUs present in at least 50% of the samples, with others at 90%. A study on the seeds of *Crotalaria pumila* revealed *Methylobacterium* as the dominant OTU and constituted more than 80% of the core microbiome [26]. The genus *Pseudomonas* is ubiquitous in nature and part of the core endomicrobiome of many plants ranging from model plants like *Arabidopsis thaliana* to medicinal plants like *Cannabis sativa* [27,28,29]. *Pseudomonas* sp. can confer unique characteristics to the host plant and are well known for plant growth promotion [22,30]. 

As with DGGE, the results of Illumina MiSeq analysis confirmed that plant tissues affected the composition, diversity and richness of endophytic bacteria in *P. colorata*. Whilst these results were congruent for both DGGE and Illumina MiSeq, the data for the richness of endophytic bacteria was in contrast to both the techniques. According to the DGGE data, leaves had a higher richness of endophytic bacterial communities compared to stems and roots as opposed to Illumina where roots had higher richness compared to leaves and stems. This highlights some disadvantages of DGGE where different taxa can co-migrate in the same band and only the abundant taxa are visualized and the only way to determine the identity is to sequence all the bands which are both time consuming and difficult given how close the bands are [31,32]. 

The endomicrobiome may be involved in providing protection against pathogens either directly through antagonism or indirectly by influencing host biochemical pathways and the production of secondary metabolites [33]. PICRUSt analysis showed that some of the endophytic bacteria of *P. colorata* may be involved in the production of bioactive secondary metabolites. Comparison of the predicted gene functions in *P. colorata* revealed that the endophytic bacteria within the tissues were associated with different metabolic activities like metabolism of carbohydrates and amino acids, which could help with the penetration of root cell walls and aid in colonization [4,34]. Similar work using PICRUSt in *Brachypodium distachyon* revealed gene categories related to metabolism, genetic information processing, cell motility and membrane transport [35]. 

This study is the first to describe the isolation and biocontrol potential of culturable bacterial endophytes from *P. colorata*. All the tissues sampled (roots, stems and leaves) hosted at least one culturable endophyte. These results support the theory that all the individual plants on earth are colonized by one or more endophyte [36]. The number of endophytic bacteria isolated in this study were comparable to other studies. For example, similar studies on the medicinal plants *Ferula songorica* (Chinese medicinal plant) and *L. scoparium*, respectively, isolated 170 and 192 culturable endophytic bacteria [7,21]. Leaves of *P. colorata* yielded the lowest number of culturable bacterial endophytes (6.17%, *n* = 25). The low number of culturable endophytes from the leaves of *P. colorata* could be because they contain the sesquiterpene dialdehyde polygodial which is known to have very strong activity against bacteria and fungi [13,14]. 

The leaves of *P. colorata* contain spherical oil vesicles called idioblasts, which were likely the sites of polygodial biosynthesis and storage [37]. During the recovery of endophytes, after dissecting the leaf, the endophytic bacteria may have been killed due to the direct contact with polygodial from ruptured idioblasts. From the total endophytic bacteria tested (*n* = 405), 9.2% (*n* = 37), 11.4% (*n* = 46), 8.0% (*n* = 32), 8.9% (*n* = 36) bacterial endophytes showed antagonistic activity against *Neofusicoccum luteum*, *N. parvum*, *I. liriodendri* and *Neonectria ditissima*, respectively. Some of the isolates showed high activity against phytopathogenic fungi indicating their potential as biocontrol agents.

*Bacillus* sp. TP1LA1B and *Pantoea* sp. AP1SA1 solubilized phosphate, secreted siderophores in vitro (data not shown) also increased the shoot dry weight, height and number of internodes in *P. colorata* seedlings. Studies have demonstrated that members of these genera can improve plant growth and overall fitness via the production of phytohormones, siderophores and organic acids that are involved in the solubilization of phosphate [38,39].

In conclusion, this study for the first time describes the structure of the bacterial endomicrobiome of the primitive angiosperm *P. colorata*. A core endomicrobiome that was tissue-specific was revealed for the first time. The identification of a core endomicrobiome suggests that the endophytes of *P. colorata* are likely to be important and involved in the physiological processes of the host. In addition, *P. colorata* contains several culturable endophytic bacteria with antimicrobial properties, some of which were able to improve the growth of the host plant. Future studies could further identify members which may play an important role in the protection of the host plant and chemistry.

## 4. Materials and Methods 

### 4.1. Sample Collection and Processing

A total of 87 individual *P. colorata* plants were sampled from ten distinct locations in the North and South Island of New Zealand (Table 5). 

Leaf, stem and root samples from healthy *P. colorata* were collected between March and August 2014. The plants and tissues collected were stored in a refrigerator and processed within 3 days from the time of sampling. The *P. colorata* tissues were surface sterilized using the 5-step sterilization method [21]. The surface-sterilized tissues were cut into 1-mm wide portions and plated onto R2A agar (Difco) amended with nystatin and cycloheximide (50 μg/mL) to prevent the growth of fungi [40]. The plates were incubated at 25 °C in total darkness for 3–5 d. Emerging colonies were sub-cultured onto nutrient agar (NA, Difco) plates. Small sections of surface-sterilized *P. colorata* tissues were used for extracting DNA for DGGE and Illumina MiSeq. 100 μL of water from the final surface sterilization wash was plated onto R2A agar and leaves were also imprinted onto R2A agar and incubated at 25 °C for 24–48 h to check if the surface sterilization process was effective. 

### 4.2. Diversity Analysis of the Endophytic Bacteria in P. colorata Using DGGE

To avoid extraneous DNA from epiphytic microbes being amplified by PCR, 1.25 μL of 20 mM propidium monoazide (PMA) was added to the surface-sterilized *P. colorata* tissues prior to DNA extraction [21,41]. DNA was extracted and amplified using group-specific primers for Alphaproteobacteria, Betaproteobacteria, and Gammaproteobacteria [31,42,43] (Table S4). The amplified PCR products were separated in DGGE gels in a Cipher DGGE Electrophoresis system (CBS Scientific). The microbial communities were analyzed using Phoretix 1D Pro Gel Analysis (Totallab, UK), and the statistical analysis was performed as described previously [21,44].

### 4.3. Illumina MiSeq Metabarcoding of Bacterial Endophytes of P. colorata

For Illumina MiSeq, the composite DNA samples were prepared by pooling the DNA extracted from the same tissue type of multiple individual plants collected at the same site. In total, 31 *P. colorata* tissue samples (leaves, stems and roots) representing 10 sites across New Zealand were obtained by pooling the DNA from 87 individual plants (Table S5). The V3-V4 hypervariable region of the 16S rRNA gene of *P. colorata* endophytic bacteria were amplified using the primers 341F (5’–CCTACGGGNGGCWGCAG-3’) and 805R (5’-GACTACHVGGGTATCTAATCC-3’ [45]. The PCRs were performed in a total volume of 25 μL and contained 12.5 μL of 2 × KAPA HiFi HotStart ReadyMix (Kapa Biosystem, South Africa), 5 μL each of the forward and reverse primer stock (1 μM) and 2.5 μL of genomic DNA at a concentration of 5 ng/μL. The resulting libraries were quantified using the Qubit DNA ds BR assay system (Thermo-Fisher Scientific, USA) as per the manufacturer’s protocol. Amplicon libraries were sequenced by New Zealand Genomics Ltd using the Illumina MiSeq v2 platform (250 bp paired-end). The generated reads were analyzed using QIIME 1.8.0 (Table S6).

### 4.4. Functional Prediction of P. colorata Bacterial Endomicrobiome using PICRUSt 

To predict the possible functions of bacterial endophytes in *P. colorata*, an open-source tool called PICRUSt (http://picrust.github.com) was used [46]. PICRUSt uses 16S rRNA abundances to predict the gene families. Prior to using the function prediction analysis in PICRUSt, the abundances of different 16S rRNA genes were normalized based on the known gene copy number for that OTU.

### 4.5. Biocontrol Activity against Phytopathogenic Fungi 

Bacterial endophytes recovered from *P. colorata* were screened for their ability to inhibit the growth of *Neofusicoccum luteum* ICMP 16678, *Neofusicoccum parvum* MM562, *Ilyonectria liriodendri* WPa1c and *Neonectria ditissima* ICMP 14417 in dual culture assays [21]. All the experiments were conducted in triplicates using appropriate control plates. The presence of an inhibition zone was recorded as a positive activity and based on the inhibition zone size, the activity was further classified as high, moderate and low activity [21,44].

### 4.6. Identification of Bioactive Bacteria by Sequencing the 16S rRNA Gene 

Bacterial isolates that showed the highest activity against phytopathogenic fungi tested were identified by sequencing the 16S rRNA gene. PureGene kit (Qiagen) was used to extract DNA, which was amplified using the primer pair F27 (5’-AGA GTT TGA TCM TGG CTC AG-3’), R1494 (5’-CTA CGG YTA CCT TGT TAC GAC-3’) [47,48]. The PCR-amplified 16S rRNA region was sequenced directly at the Lincoln University Sequencing Facility (Applied Biosystems 3130xl Genetic Analyzer). Ambiguous regions of the sequences were trimmed using DNAMAN v4 (Lynnon Biosoft, Canada) and compared using NCBI BLAST (basic local search alignment tool) and the GenBank database. 

### 4.7. Effect of Endophytic Bacterial Inoculants on P. colorata Seedlings

The influence of select endophytic bacteria on the growth of *P. colorata* was assessed by inoculating six-week-old *P. colorata* seedlings in the glasshouse. The plants were sourced from Southern Woods Plant Nursery (Christchurch, New Zealand) and did not have a well-formed root system at the time of purchase. The seedlings were acclimatized in Lincoln University shade house for approximately 1 month (February 2017). After the seedlings were established, they were transferred into 1 L pots with potting mix and arranged in a complete randomized block design with each treatment having 10 replicates. The endophytic bacterial inoculants of AP1SA1 and TP1BA1B were prepared in nutrient broth (NB, Difco) and adjusted to 10^5^ to 10^6^ cells/ mL. The treatments were applied as root drenches, where 50 mL of the respective cell suspension was added to the root region of *P. colorata* seedlings [49]. Sterile distilled water without any cell suspension was added to the control seedlings. Prior to setting up the experiments, the shoot length and stem girth were measured using a digital caliper. 24-48 hours prior to inoculation (March 2017) with the endophytic bacteria the seedlings did not receive any water. 24 hours post-inoculation, the seedlings were watered once every day and the plant health was monitored on a regular basis. Three months after inoculation (May 2017), the seedlings were treated again with spore suspensions of their respective treatments. One month (June 2017) after the second inoculation, the seedlings were destructively harvested. The shoot height, number of internodes, shoot and root dry weight for each plant were measured and the data were analyzed using Minitab 17 (Lead Technologies, Australia) as described previously [44]. 

## Figures and Tables

**Table 1:** Influence of sampling location and tissue on the *P. colorata* endophytic bacterial communities similarity (A) and richness (B).

Factors	Alphaproteobacteria		Betaproteobacteria		Gammaproteobacteria	
	A	B	A	B	A	B
Location	0.323	0.036 *	0.149	0.756	0.312	0.204
Plant tissue	0.001 **	<0.001 **	0.001 **	<0.001 **	0.001 **	<0.001 **
Location vs plant tissue	0.021 *	0.253	0.001 **	0.057	0.100	0.164

* significant difference (*P* ≤ 0.05), ** highly significant difference (*P* ≤ 0.005) of *P. colorata* endophytic bacterial communities similarity based on PERMANOVA and microbial richness based on GLM (generalized linear model).

**Table 2:** Activity of select endophytic bacteria isolated from *P. colorata* against fungal phytopathogens. Activity was assessed as high activity (+++) where growth was completely inhibited, moderate activity (++), low activity (+), no activity (-).

	Phytopathogenic Fungi			
Isolate	*Neofusicoccum luteum*	*Neofusicoccum parvum*	*Ilyonectria liriodendri*	*Neonectria ditissima*
TP1LA1B	+++	++	++	++
TP1LC1B	+++	++	++	++
TOYPRB1R	+++	++	++	++
KIP1SB1B	+++	++	++	++
KRP1BA1	+++	+++	++	++
AP1SA1	+++	+++	-	-
KRP1BC1	+++	+++	-	-
KRP1BB1	+++	+++	++	++
KRP1BA2	+++	+++	++	++
KVP1BC1	+++	++	-	-

**Table 3:** Identity of endophytic bacteria from leaf, stem and root tissue based on 16S rRNA sequencing.

Isolate	Tissue	Reference Strain (GenBank)	Query Cover (%)	Similarity (%)	Accession no.
TP1LA1B	Leaf	*Bacillus amyloliquefaciens* strain ML471	99	99	KC692205
TP1LC1B	Leaf	*Bacillus subtilis* strain Y5	100	99	GQ148816
TOYPRB1R	Root	*Bacillus subtilis* strain AU04	99	98	MF590152
KIP1SB1B	Stem	*Bacillus* sp. strain A3	99	99	KU904495
KRP1BA1	Stem	*Pseudomonas fluorescens* strain 4G628	100	99	KY939748
KRP1BA2	Stem	*Pseudomonas fluorescens* strain 4G628	100	99	KY939748
AP1SA1	Stem	*Pantoea* sp. ATY73	100	98	HQ219992
KRP1BC1	Stem	*Pseudomonas* sp. strain PCH123	99	98	MF774109
KRP1BB1	Stem	*Pseudomonas* sp. ps10-15	98	98	AY303256
KVP1BC1	Stem	*Erwinia* sp. strain ES1	99	98	KY446019

**Table 4:** Influence of endophytic bacterial treatments on the growth of *P. colorata* seedlings.

Endophytic Bacteria	Shoot Height (cm)	Dry Weight (g)		Number of Internodes
		Shoot	Root	
*Pantoea* sp. AP1SA1	5.79 a ^1^	0.79 bc	0.47 b	6.7 ab
*Bacillus* sp. TP1LA1B	5.70 a	1.38 a	0.69 a	6.8 a
Control	3.12 b	0.76 b	0.46 b	3.7 c
P Value	<0.005	<0.005	<0.05	<0.001
LSD (5%)	1.63	0.24	0.17	0.49

^1^ No significant difference for means followed by the same letter based on LSD at *P* = 0.05.

**Table 5:** *Pseudowintera colorata* sampling sites across New Zealand.

Sampling Site	Latitude (°South)	Longitude (°East)	Region	North/South Island	Plant Maturity
Taihape Scenic Reserve	−39.67635	175.80560	Manawatu-Wanganui	North Island	Mature
Tongariro National Park	−39.02237	175.71810	Manawatu-Wanganui	North Island	Mature
Kaimanawa Forest Park	−38.94721	175.94370	Manawatu-Wanganui	North Island	Mature
Lake Rotopounamu Scenic Reserve	−39.02656	175.73502	Manawatu-Wanganui	North Island	Mature
Kahurangi National Park	−41.07224	172.59166	Nelson/Tasman	South Island	Mature
Paringa Forest	−43.69379	169.40724	West Coast	South Island	Mature and Immature
Arthur’s Pass National Park	−42.94215	171.56414	Canterbury	South Island	Mature
Kaituna Valley Scenic Reserve	−43.71655	172.7554	Canterbury	South Island	Mature and Immature
Peel Forest	−43.91835	171.25934	Canterbury	South Island	Mature and Immature
Otago Peninsula Scenic Reserve	−45.88184	170.58049	Otago	South Island	Mature

## Supplementary Materials

The following are available online at https://www.mdpi.com/2223-7747/9/2/156/s1, Table S1: Permanova and General Linear Model (Anova) result of the influence of tissue type, location and interactions between tissue type and location on endophytic Alphaproteobacteria communities similarity and richness; Table S2: Permanova and General Linear Model (Anova) result of the influence of tissue type, location and interactions between tissue type and location on endophytic Betaproteobacteria communities similarity and richness; Table S3: Permanova and General Linear Model (Anova) result of the influence of tissue type, location and interactions between tissue type and location on endophytic Gammaproteobacteria communities similarity and richness; Table S4: Sequence details of group-specific 16S rRNA primers used for PCR; Table S5: Pooled concentration of DNA used for Illumina MiSeq sequencing for the different samples; Table S6: Script used in Qiime 1.8.1 for Illumina MiSeq data analysis.
