# Supplementary material for: Community Structure, Diversity and Potential of Endophytic Bacteria in the Primitive New Zealand Medicinal Plant Pseudowintera colorata
Source: Plants (Basel). 2020 Jan 27;9(2):156. doi: 10.3390/plants9020156 (PMC7076676; doi:10.3390/plants9020156)
Supplement: Supplementary file 1 [file plants-09-00156-s001.pdf]

Supplementary Materials

Table S1: Permanova and General Linear Model (Anova) result of the influence of tissue type, location and interactions between tissue type and location on the similarity and richness of endophytic Alphaproteobacteria communities in *P. colorata*

**Tissue**						
Source	DF	SS	MS	Pseudo-F	P(perm)	perms
Ti	2	35635	17817	7.0549	0.001	999
Res	43	1.086E+05	2525.5			
Total	45	1.4423E+05				

**Location**						
Source	DF	SS	MS	Pseudo-F	P(perm)	perms
Lo	9	30482	3386.9	1.0719	0.283	997
Res	36	1.1375E+05	3159.7			
Total	45	1.4423E+05				

**Tissue*Location**						
Source	DF	SS	MS	Pseudo-F	P(perm)	perms
Ti	2	27938	13969	6.6545	0.001	998
Lo	9	24382	2709.1	1.2905	0.076	997
TixLo**	14	38625	2759	1.3143	0.021	999
Res	19	39884	2099.2			
Total	44	1.4039E+05				

One-way ANOVA: Number of Bands versus Tissue

Analysis of Variance

**Tissue**					
Source	DF	SS	MS	F-Value	P-Value
Tissue	2	575.8	287.910	32.77	0.000
Error	44	386.6	8.787		
Total	46	962.4			

Means

Tissue	N	Mean	StDev	95% CI	Grouping
Leaf	17	11.235	3.882	(9.786, 12.684)	A
Root	13	5.615	2.181	(3.959, 7.272)	B
Stem	17	3.176	2.351	(1.728, 4.625)	C

Pooled StDev = 2.96420

Means that do not share a letter are significantly different.

One-way ANOVA: Number of Bands versus Location

**Location**					
Source	DF	SS	MS	F-Value	P-Value
Location	9	177.0	19.69	1.66	0.122
Error	51	602.7	11.818		
Total	60	1164.9			

General Linear Model: Number of Bands versus Tissue, Location

**Tissue*Location**					
Source	DF	SS	MS	F-Value	P-Value
Tissue*Location	18	440.5	24.470	1.31	0.253
Error	28	522.0	18.642		
Total	46	962.4			

Table S2: Permanova and General Linear Model (Anova) result of the influence of tissue type, location and interactions between tissue type and location on the similarity and richness of endophytic Betaproteobacteria communities in *P. colorata*

**Tissue**						
Source	DF	SS	MS	Pseudo-F	P(perm)	perms
Ti	2	28213	14107	4.8161	0.001	999
Res	50	1.4645E+05	2929			
Total	52	1.7466E+05				

**Location**						
Source	DF	SS	MS	Pseudo-F	P(perm)	perms
Lo	10	37632	3763.2	1.1534	0.149	996
Res	42	1.3703E+05	3262.7			
Total	52	1.7466E+05				

**Tissue*Location**						
Source	DF	SS	MS	Pseudo-F	P(perm)	perms
Ti	2	29770	14885	7.3893	0.001	998
Lo	10	41613	4161.3	2.0658	0.001	999
TixLo**	16	56778	3548.6	1.7616	0.001	998
Res	24	48346	2014.4			
Total	52	1.7466E+05				

One-way ANOVA: Number of Bands versus Tissue

Analysis of Variance

**Tissue**					
Source	DF	SS	MS	F- Value	P-Value
Tissue	2	639.6	319.78	14.85	0.000
Error	49	1055.1	21.53		
Total	51	1694.7			

Means

Tissue	N	Mean	StDev	95% CI	Grouping
Leaf	22	4.909	3.421	(2.921, 6.897)	B
Root	14	7.71	6.34	(5.22, 10.21)	B
Stem	16	13.19	4.37	(10.86, 15.52)	A

Means that do not share a letter are significantly different.

Fisher Individual 95% CIs

General Linear Model: Number of Bands versus Location

**Location**					
Source	DF	SS	MS	F- Value	P-Value
Location	9	132.8	14.75	0.64	0.756
Error	40	922.3	23.06		
Total	49	1694.7			

General Linear Model: Number of Bands versus Tissue*Location

**Tissue*Location**					
Source	DF	SS	MS	F- Value	P-Value
Tissue*Location	18	552.6	30.70	1.89	0.057
Error	31	502.5	16.21		
Total	51	1694.7			

Table S3: Permanova and General Linear Model (Anova) result of the influence of tissue type, location and interactions between tissue type and location on the similarity and richness of endophytic Gammaproteobacteria communities in *P. colorata*

**Tissue**						
Source	DF	SS	MS	Pseudo-F	P(perm)	perms
Ti	2	32025	16012	7.3321	0.001	998

Res	49	1.0701E+05	2183.9			
Total	51	1.3903E+05				

**Location**						
Source	DF	SS	MS	Pseudo-F	P(perm)	perms
Lo	9	25983	2887	1.0726	0.312	996
Res	42	1.1305E+05	2691.7			
Total	51	1.3903E+05				

**Tissue*Location**						
Source	DF	SS	MS	Pseudo-F	P(perm)	perms
Ti	2	30934	15467	8.281	0.001	999
Lo	9	22444	2493.8	1.3352	0.074	999
TixLo**	16	36226	2264.1	1.2122	0.1	998
Res	24	44827	1867.8			
Total	51	1.3903E+05				

One-way ANOVA: Number of Bands versus Tissue

Analysis of Variance

**Tissue**					
Source	DF	SS	MS	F-Value	P-Value
Tissue	2	160.6	80.322	19.79	0.000
Error	45	182.7	4.059		
Total	47	343.3			

Means

Tissue	N	Mean	StDev	95% CI	Grouping
Leaf	22	6.455	1.845	(5.589, 7.320)	B
Root	14	10.643	2.590	(9.558, 11.727)	A
Stem	12	7.000	1.477	(5.829, 8.171)	B

**Location**					
Source	DF	SS	MS	F-Value	P-Value
Location	9	48.59	5.399	1.45	0.204
Error	36	134.08	3.724		
Total	47	343.31			

**Tissue*Location**					
Source	DF	SS	MS	F-Value	P-Value
Tissue*Location	18	91.48	5.082	1.50	0.164
Error	27	91.19	3.377		
Total	45	343.31			

Table S4: Sequence details of the group-specific 16S rRNA primers used for PCR

Group	Primer	Sequence (‘5 - 3’)
α-Proteobacteria	F203α	CCG CAT ACG CCC TAC GGG GGA AAG ATT TAT
	L1401	CGG TGT GTA CAA GAC CC
	341F-GC	GC–CCT ACG GGA GGC AGC AG
	518R	ATT ACC GCG GCT GG
β-Proteobacteria	Beta359F	GGG GAA TTT TGG ACA ATG GG
	Beta682R	GGG GAA TTT TGG ACA ATG GG
	518F-GC	GC–CCA GCA GCC GCG GTA AT
γ-Proteobacteria	Gamma395F	CMA TGC CGC GTG TGT GAA
	Gamma871R	ACT CCC CAG GCG GTC DAC TTA
	518F-GC	GC–CCA GCA GCC GCG GTA AT
	785R	CTA CCA GGG TAT CTA ATC C

Table S5: Pooled concentration of DNA used for Illumina MiSeq sequencing for the different samples

**Site**	**Site code**	**Tissue**	**Concentration ng/mL**
Arthur's Pass	AP	Leaf	13.5
		Stem	15.2
		Root	16.5
Kiko Road	KI	Leaf	21.0
		Stem	15.9
		Root	13.3
Tongariro Nat. Park	TO	Leaf	12.1
		Stem	12.4
		Root	12.7
Taihape Reserve	TP	Leaf	12.4

		Root	12.6
		Stem	8.14
Lake Rotopounamu	RO	Stem	11.1
		Stem	8.05
		Leaf	7.38
Otago	OT	Leaf	19.1
		Stem	12.1
Kahurangi Nat. Park	KH	Leaf	12.4
Peel Forest	PL	Leaf	15.8
		Stem	7.13
		Root	18.4
Paringa	PR	Leaf	12.8
		Stem	8.06
		Root	15.4
	PRY 1	Leaf	12.6
	PRY 2	Leaf	26.3
	PRY 3	Leaf	35.3
Kaituna Valley	KV	Leaf	27.8
		Stem	22.9
		Root	14.0
	KVY	Leaf	35.6

Table S6: Script used in QIIME 1.8.1 for Illumina MiSeq data analysis

1. Join paired end with fastq join

join_paired_ends.py -f forward_reads.fastq -r reverse_reads.fastq -o joined.fastq

2. Quality (Phred 15 and maxee 0.5) and length (400 bp) trimming of fastq file

-fastq_filter joined.fastq -fastq_trunclen 400 -fastq_truncqual 15 -fastq_maxee 0.5 -fastqout filtered.fastq

3. Combined fastq data from two Illumina Miseq runs

filteredrun1.fastq filteredrun2.fastq >filteredjoin.fastq

4. Converting to fasta from fastq file

fastq_to_fasta -i filtered.fastq -o filteredjoin.fasta

5. Combine all fasta file and add label according mapping file

add_qiime_labels.py -i Allfastafile -m mappingfile.txt -c combined_seqs.fasta

6. Pick OTUs, assign taxonomy, and create an OTU table against a reference set of OTUs

pick_closed_reference_otus.py -i combined_seqs.fasta -o otus/

7. Remove OTU belonged to chloroplast and Mitochondria from OTU table

filter_taxa_from_otu_table.py -i otu.biom -o otunonplant.biom -n c__Chloroplast,f__mitochondria

8. Run alpha and beta diversity analysis

core_diversity_analyses.py -o alldataset/ -i otunonplant.biom -m mappingfile.txt -t rep_tree.tre -e 448

9. Identify the core OTUs in otu_table.biom, defined as the OTUs that are present in at least 80% of the samples.
compute_core_microbiome.py -i otunonplant.biom -otu_table_core.biom
